# Dissociation protocols influence the phenotypes of lymphocyte and myeloid cell populations isolated from the neonatal lymph node

**DOI:** 10.3389/fimmu.2024.1368118

**Published:** 2024-05-01

**Authors:** Jarina P. DaMata, Amanda E. Zelkoski, Paula B. Nhan, Katherine H. E. Ennis, Ji Sung Kim, Zhongyan Lu, Allison M. W. Malloy

**Affiliations:** ^1^ Laboratory of Infectious Diseases and Host Defense, Department of Pediatrics, Uniformed Services University of Health Sciences (USUHS), Bethesda, MD, United States; ^2^ Henry M. Jackson Foundation for the Advancement of Military Medicine, Inc., Bethesda, MD, United States

**Keywords:** neonatal mouse, lymph node, single-cell suspension, mechanical dissociation, enzymatic digestion, myeloid cells, lymphocytes

## Abstract

Frequencies and phenotypes of immune cells differ between neonates and adults in association with age-specific immune responses. Lymph nodes (LN) are critical tissue sites to quantify and define these differences. Advances in flow cytometry have enabled more multifaceted measurements of complex immune responses. Tissue processing can affect the immune cells under investigation that influence key findings. To understand the impact on immune cells in the LN after processing for single-cell suspension, we compared three dissociation protocols: enzymatic digestion, mechanical dissociation with DNase I treatment, and mechanical dissociation with density gradient separation. We analyzed cell yields, viability, phenotypic and maturation markers of immune cells from the lung-draining LN of neonatal and adult mice two days after intranasal respiratory syncytial virus (RSV) infection. While viability was consistent across age groups, the protocols influenced the yield of subsets defined by important phenotypic and activation markers. Moreover, enzymatic digestion did not show higher overall yields of conventional dendritic cells and macrophages from the LN. Together, our findings show that the three dissociation protocols have similar impacts on the number and viability of cells isolated from the neonatal and adult LN. However, enzymatic digestion impacts the mean fluorescence intensity of key lineage and activation markers that may influence experimental findings.

## 1 Introduction

Lymph nodes (LNs) are small organs present throughout the mammalian body that promote the interaction of antigen-presenting cells (APCs), lymphocytes and other immune cells, leading to activation of the adaptive immune response (1–4). Analysis of these tissue sites provides important opportunities to define mechanisms of immune regulation. Within the LN, APCs engage and activate T and B cells to influence the generation of both acute (5, 6) and memory (7) responses that clear infection and resolve tissue damage (8, 9) or promote pathologic inflammation (9, 10). The balance of homeostasis (11) and inflammatory responses (12) is regulated via differential expression of costimulatory molecules and production of cytokines by APCs and accurate measurement of these molecules as well as the cells that supply them inform our understanding of immunity. Between the complex landscape of APCs, conventional dendritic cells (cDCs) have been shown to specialize in activation of naïve T cells (6, 13–15), while plasmacytoid dendritic cells (pDCs) produce type I interferons, a group of cytokines crucial in antiviral response (16–19). In addition, subsets of macrophages support both the architecture of the LN as well as capture antigen to increase (20, 21) or dampen the immune response (22). Immune engagement differs in early life compared to adulthood and has been shown in both mice (23–25) and humans (26–30). External factors such as bacterial exposure (31) and colonization (32), as well as internal factors such as organ development and growth (33, 34), are thought to influence immune development. Important questions remain regarding how age (35, 36) and exposure (12, 37) influence the diverse subsets and functions of APCs and their impact on adaptive immunity (37–39).

Advances in technologies such as spectral flow cytometry, mass cytometry and single-cell RNA sequencing provide new opportunities to study the complexity of age-dependent immunity (27, 40–42). These technologies require analysis of single-cell suspensions, and disrupting the architecture of the LN can have consequences for immunologic measurements. LN architecture continues to develop after birth (43) and protocols for producing single-cell suspensions have not previously taken this into consideration. Specialized subsets of endothelial cells and reticular cells connected by adhesion molecules and collagen support the structure of the LN (44). Different approaches to disrupt these structures have been published and further understanding regarding the effect of these protocols on cell isolation, phenotypic marker recognition, and expression of activation molecules is required to determine how these protocols impact study outcomes.

Dissociation of LN tissue with mechanical force is used in most protocols and can take many forms (45, 46). Our group has previously published on the combined use of tension and compression force by compressing LN between the frosted ends of two slides and sliding them back and forth both compressing and pulling the LN apart (13, 24, 26, 47). Other protocols have used shearing (46) and tension (45) forces by using scissors or needles to cut and pull the LN apart. Cutting or teasing the LN results in small particles of tissue that are then enzymatically digested to further release immune cells. Enzymatic digestion is considered essential for mucosal tissues in order to isolate sufficient numbers of immune cells (48–51). Depending on the types of cells under investigation and the tissue site enzymes can be used to disrupt collagen, extracellular matrix, adhesion molecules and cell-to-cell junctions (52). However, common enzymes including dispase, types of collagenase (48, 51, 53, 54) and trypsin have been associated with alterations of phenotypic markers used in flow cytometry. Single-cell RNA sequencing of dissociated tissues has also indicated that temperature and duration of collagenase treatment can alter gene expression (55, 56). After tissue dissociation, protocols may enrich for cells of interest through methods such as magnetic bead isolation, fluorescence-activated cell sorting (FACS), or density gradient separation, all of which may contribute to deliberate or inadvertent cell loss (57–60).

In this study, we aimed to evaluate the effect of three dissociation methods for generating single-cell suspensions from lung-draining LNs (dLN) in neonatal mice for use in spectral flow cytometric analysis. These methods include enzymatic digestion with collagenase D (45), mechanical dissociation with DNase I, and mechanical dissociation followed by density gradient separation (24, 26, 47, 61). To evaluate the protocols, we analyzed the frequencies and cell counts of a broad variety of LN-resident and non-resident immune cell populations based on flow cytometric measurements of 25 phenotypic and activation molecules. No differences were observed in the cell counts of conventional dendritic cells and macrophage populations upon comparison of the enzymatic and mechanical protocol for dissociating the neonatal LN. However, the enzymatic protocol led to reduced mean fluorescence intensity (MFI) of the pan-B cell marker B220 and CXCR5. Additionally, activation markers such as CD86, were increased in the neonatal cells processed with the enzymatic versus mechanical protocols. Our findings identify phenotypic markers altered by collagenase D and key activation markers that may be upregulated, potentially influencing the interpretation of experimental results.

## 2 Materials and methods

### 2.1 Animals

Adult (7- to 10-week-old) female CB6F1/J mice were purchased from The Jackson Laboratories (Bar Harbor, ME, USA). CB6F1/J neonatal mice (6-day old) were obtained by in-house breeding of C57BL/6 males and BALB/c females (The Jackson Laboratories) (62). Mice were bred and housed in specific-pathogen free conditions at an American Association for the Accreditation of Laboratory Animal Care-accredited animal facility at the Uniformed Services University (USU) and housed in accordance with the procedures outlined in the Guide for the Care and Use of Laboratory Animals, National Research Council, 8th Edition, 2011. This study was conducted under USU Institutional Animal Care and Use Committee (IACUC) approval (protocol# PED-23-908).

### 2.2 Infection and tissue collection

The A2 strain of RSV was propagated in HEp-2 cells (ATCC, Manassas, VA, USA) as previously described (63). Mice were anesthetized using aerosolized isoflurane (3%) inhalation. Anesthetized mice were intranasally infected with 2 × 10^6^ plaque forming units (PFU) of live RSV. Volume of infection was adjusted for 5 μl/g of weight in neonatal mice or 100 μl per adult mouse by dilution with sterile PBS. All infections were performed in a class II biosafety cabinet with appropriate PPE and BSL2 safety procedures. At two days post infection (2dpi), mice were euthanized via intraperitoneal injection of Euthasol pentobarbital solution (Virbac, Carros, France). Lung-draining LNs (dLN) were isolated from all animals as previously described (24).

### 2.3 Lymph node dissociation

#### 2.3.1 Enzymatic protocol

Prior to isolation of lung-draining LNs, 10 mg/mL collagenase D (Roche, Basel, Switzerland) was diluted to 1 mg/mL using HBSS (approximately 0.15 Wünsch U/mL or 250 collagen digestion units (CDU)/mL). FBS pretreated with 0.02 mM of EDTA was added to the solution at 1%. The solution was aliquoted 2 mL into each well of a 6-well cell culture plate and placed on ice for the duration of LN isolation. Isolated dLNs were transferred into individual wells. After isolation, LNs were teased apart using two 26 ½ G needles each attached to 1 mL syringes. Samples were teased until remaining material could not be further dissociated. Cells were incubated at 37°C for 30 min to allow for collagenase activity. After incubation, 100 µL of 100mM EDTA per mL of 1 mg/mL collagenase D was added to each well and incubated at 37°C for 5 min to terminate collagenase activity. Cells were then filtered through a 70-µm nylon filter into a 35 × 10 mm petri dish and the plunger of a 3 mL syringe was used to assist, as in the mechanical with DNase I protocol. The plunger, petri dish, and nylon filter were washed with a total of 3 mL HBSS. The volume was transferred to a 15 mL conical and washed with HBSS. An additional wash with HBSS was performed to remove remaining collagenase. Cells were resuspended in PBS containing 2% FBS.

#### 2.3.2 Mechanical with DNase I protocol

Isolated LNs were transferred into a polystyrene tube containing RPMI (Fisher Scientific, Waltham, MA, USA) with 10% FBS (GeminiBio, West Sacramento, CA, USA) (R10) at room temperature (RT) until completion of LN collection. Suspended LNs were transferred to 35 × 10 mm petri dishes and mechanically dissociated using the frosted sides of two microscope slides. To dissociate, the dLN was placed on one rough edge of one slide and the rough edge of the second slide was moved gently in a circular motion to break down the tissue. Slides and petri dishes were washed with R10 for a final volume of 3 mL. DNase I (Roche, Basel, Switzerland) was added for a final concentration of 10 µg/mL and cells were incubated at 37°C for 25 min. After incubation, cells were filtered through a 70-µm nylon filter into a 35 × 10 mm petri dish. The plunger of a 3 mL syringe was used to gently mash residual tissue through the filter. The plunger, petri dish, and nylon filter were washed with HBSS. The single-cell suspension was transferred to a 15 mL conical and then washed with PBS containing 2% FBS.

#### 2.3.3 Mechanical with Ficoll gradient separation protocol

Isolated LNs were transferred into a polystyrene tube containing R10 at RT until completion of LN collection. Suspended LNs were transferred to 35 × 10 mm petri dishes and mechanically dissociated as in the mechanical with DNase I protocol. Slides and petri dishes were washed with R10 for a final volume of 3 mL. Volume was gently layered onto Fico/Lite-LM (R&D Systems, Minneapolis, MN, USA) at a 1:1 ratio and centrifuged for density separation at 1250 G for 20 min at RT with no brake. Using a transfer pipette, the mononuclear cell layer was transferred into a 15 mL conical containing PBS. Single-cell suspensions were washed with PBS containing 2% FBS.

### 2.4 Surface staining and spectral flow cytometry

Prior to staining, cells were treated with anti-mouse CD16/CD32 (BD Biosciences, San Jose, CA, USA) in PBS containing 2% FBS at 4°C for 10 min. Cells were then stained with a mix of the fluorochrome-conjugated antibodies (**Supplementary Table 1**) and Live/Dead fixable blue viability dye (Thermo Fisher, Waltham, MA, USA) diluted in Brilliant Stain Buffer (BD Biosciences, San Jose, CA, USA) at 4°C for 20 min. Antibody amounts were determined by lot-specific titrations. Cells were washed with PBS containing 2% FBS and then fixed using BD Cytofix Fixation Buffer (BD Biosciences, San Jose, CA, USA) at 4°C for 30 min. After fixation, cells were washed with PBS containing 2% FBS. Acquisition was performed on the Cytek Aurora Spectral Analyzer (Cytek Biosciences, CA, USA) and analyzed on FlowJo (v10, BD Biosciences, San Jose, CA, USA). The cell numbers for each population were calculated by multiplying their frequency by the total live cell count obtained from automated cell counting by Cellometer Auto 2000 (Nexcelom Bioscience, Lawrence, MA, USA). Analysis with t-distributed stochastic neighbor embedding (t-SNE) was performed in FlowJo (v10, BD Biosciences, San Jose, CA, USA) by selecting and concatenating the lymphocyte subset nodes, followed by the tSNE function with the setup of iterations (1000), perplexity (100), learning rate (2000), and 25 fluorochromes in **Supplementary Table 1** excluding live/dead dye and autofluorescence. Autofluorescence signature was captured and extracted using unstained cells by the spectral flow cytometer.

### 2.5 Statistical analysis

Statistical analysis was performed using Graphpad Prism (v9, GraphPad Software Inc., Boston, MA, USA) using a one-way ANOVA, with Tukey’s multiple comparisons test. Results are expressed as means with standard error of the mean (SEM). Values of P <0.05 were considered statistically significant.

## 3 Results

### 3.1 Similar viability and cell counts were obtained from neonatal dLNs processed with different dissociation protocols

**Figure 1:**
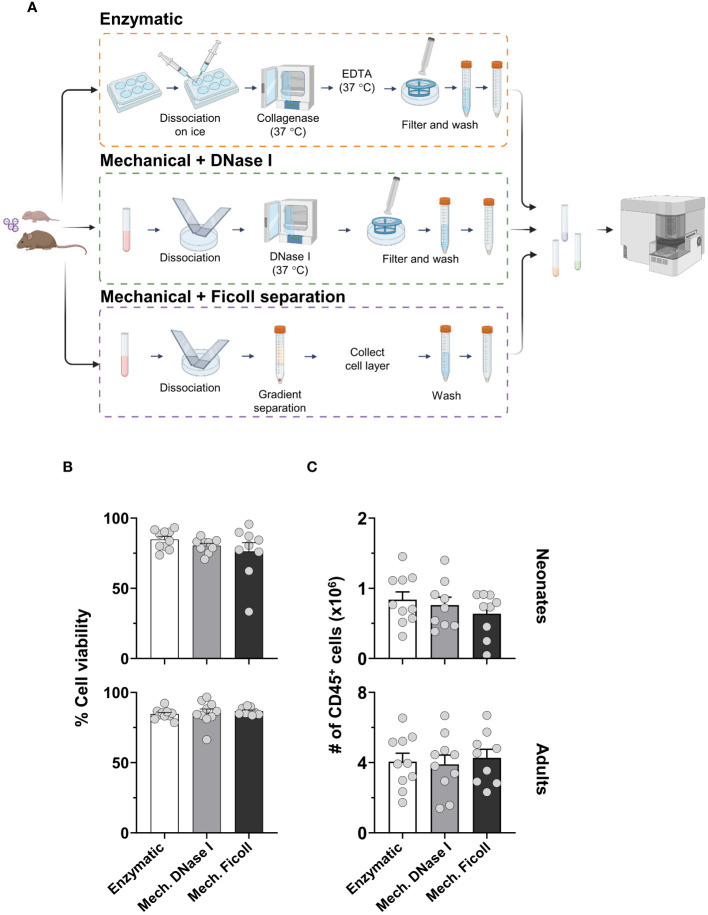
The yield and viability of isolated cells from the murine lung-draining lymph node (dLN) comparing three different dissociation protocols. **(A)** Schematic representation of protocols. **(B)** Cell viability of neonates (upper) and adults (lower) processed by the enzymatic (Enzymatic, white bars), mechanical with DNase I (Mech. DNase I, light gray bars) or mechanical with Ficoll gradient separation (Mech. Ficoll, dark gray bars) protocols. **(C)** Cell counts of CD45^+^ populations from total live cells were measured in neonatal (upper) and adult (lower) lymph nodes two days post-infection (2dpi) with RSV. Data were pooled from two independent experiments. Error bars represent standard error of the mean. Statistical analysis was performed using a one-way ANOVA Tukey’s multiple comparisons, with *P < 0.05, and **P < 0.01. Protocol pictograms were created with BioRender.com.

To evaluate the effect on immunophenotyping of the three dissociation protocols, we analyzed: i) cell viability and yields; ii) population frequency and cell counts of both migratory and resident cells; iii) the expression of phenotypic markers; and iv) activation markers. The protocols are described in detail in the methods section and illustrated in **Figure 1A**.

In the enzymatic protocol, each dLN was excessively teased with needles and incubated with collagenase D, filtered with a cell strainer, washed, and resuspended. In the mechanical with DNase I protocol (referred to as the mechanical with DNase I protocol), the dLNs were dissociated between the frosted ends of microcrope slides followed by incubation with DNase I to reduce cell clumping, but did not include collagenase D. Lastly, the mechanical with density gradient separation (referred to as the mechanical with Ficoll protocol) included dissociation of the dLN with slides. The cell suspension after mechanical dissociation was enriched for mononuclear cells by density gradient separation, washed, and resuspended.

Viability among the dissociation protocols was overall similar, on average 90% (**Figure 1B**). There was no significant difference in viability when comparing age groups by flow cytometry after removing doublets and debris (**Supplementary Figure S1A**). The total CD45-positive cell counts remained consistent across protocols (**Figure 1C**). However, the cell count from the neonatal dLN, compared to the adult dLN two days post RSV infection, exhibited a distinct difference, with approximately 3-5 times more cells quantified in the adult dLN. These findings suggest that the three protocols yielded similarly viable single-cell suspensions irrespective of age, and the cell count remained consistent between the dissociation protocols within each age group.

### 3.2 The frequencies and cell numbers of lymphocyte populations differed between the dissociation protocols

**Figure 2:**
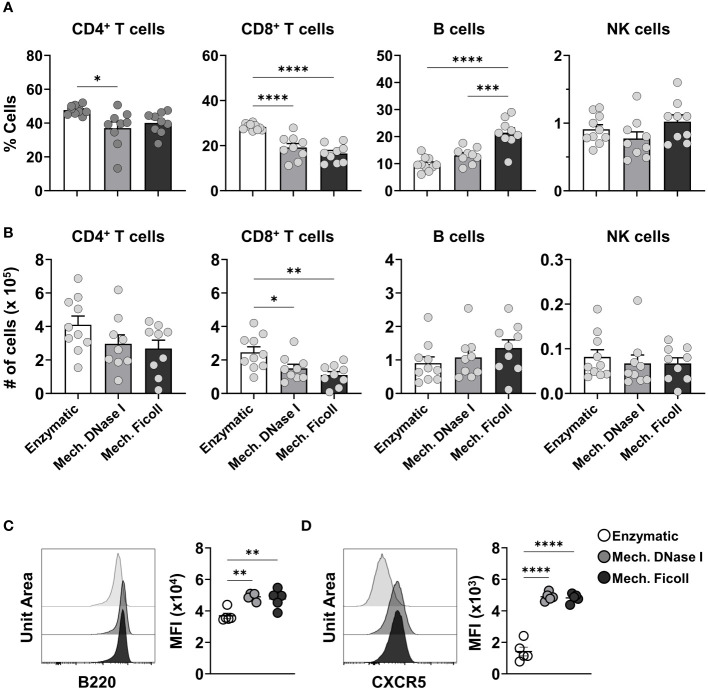
The frequencies of lymphocyte populations and B cell suface molecules differ between neonatal dLNs processed with enzymatic or mechanical protocols. **(A)** Frequencies of CD4^+^ T cells, CD8^+^ T cells, B cells and NK cells in live CD45^+^ cells from neonatal dLNs at 2 dpi processed using indicated protocols. **(B)** Cell counts of B cells, CD8^+^ T cells, CD4^+^ T cells, and NK cells from single lymph nodes. Data were pooled from two independent experiments, with 4-5 mice per experiment. **(C, D)** Representative histogram and the mean fluorescence intensity (MFI) of B220 **(C)** and CXCR5 **(D)**. MFI was representative of two independent experiments. Statistical analysis was performed using a one-way ANOVA Tukey’s multiple comparisons, with *P < 0.05, **P < 0.01, ***P < 0.005, and ****P < 0.0001. Error bars represent standard error of the mean.

Lymphocyte populations (T cells, B cells and NK cells) were identified (**Supplementary Figure S1A**) and represented the largest populations in the dLNs in both adult and neonatal mice. The frequency and cell numbers of each of these major populations in neonatal mice varied between the dissociation protocols. The enzymatic protocol yielded a higher frequency of CD4^+^ T cells, 47.7% from live CD45^+^ cells, compared to the mechanical with DNase I, or Ficoll protocols at 39% and 40.5%, respectively (**Figure 2A**). Similarly, the frequency of CD8^+^ T cells was higher after enzymatic processing. The B cell frequency was higher in samples processed with the mechanical with Ficoll protocol (21%) compared to the mechanical with DNase I (13%) and enzymatic (10%) protocols out of CD45^+^ cells. No statistical difference was observed between the dissociation protocols from the NK cell population, representing 0.9%, 0.7% and 1.0% of the live CD45^+^ cells in the enzymatic, mechanical with DNase I, or Ficoll protocol, respectively. While cell counts reflected the trends observed in frequencies, only CD8^+^ T cells exhibited significant differences between the dissociation protocols (**Figure 2B**). Lymphocyte populations measured from the dLNs of adult mice 2 days post RSV infection mirrored the trends seen in the neonatal dLNs. The CD8^+^ T cell subset frequencies were higher in the dLN following enzymatic processing compared to the mechanical with DNase I, or Ficoll protocols, while the frequency of B cells was comparatively higher in the dLN processed with the mechanical with Ficoll protocol (**Supplementary Figures S2A, B**). NK cell frequencies were slightly elevated in the enzymatic protocol (2.33% in enzymatic, 1.95% in mechanical with DNase I, and 1.76% in mechanical Ficoll protocols). Cell counts supported the frequency results, with only CD8^+^ T cells showing significant differences between the dissociation protocols.

Analysis with t-distributed stochastic neighbor embedding (t-SNE) was used to compare the fluorescence intensity of phenotypic markers identifying the lymphocyte populations (**Supplementary Figures S3A, B**). The MFI of the BUV395 anti-B220 and FITC anti-CXCR5 on B cells differed between the dissociation protocols (**Supplementary Figures S3C, D**). When analyzing B220 expression on B cells from neonatal mice, the MFI was significantly lower after processing with the enzymatic protocol (P < 0.01, **Figure 2C**). CXCR5, another surface molecule expressed on B cells, also showed a significant decrease in MFI in samples processed with the enzymatic protocol when compared to the other protocols (P < 0.0001 in both comparisons) (**Figure 2D**). The MFI reduction of B220 and CXCR5 was also observed in adults (**Supplementary Figure S3E**), indicating that it was associated with the dissociation protocol and independent of age.

These results indicate that the dissociation protocols differentially affected phenotypic and lineage markers influencing the measured frequency of lymphocyte subsets in the neonatal dLN. Similar results were found in the processed adult dLN suggesting that the differences in measured cells were reflective of the protocol rather than the age of the mouse.

### 3.3 Enzymatic digestion increased costimulatory molecules on cDC1

Next, we measured classical APC subsets. Conventional DCs (cDCs) are MHC class II and CD11c high and can be divided into cDC1 and cDC2 as they share a common precursor (39). Here we used XCR1 and CD172a to distinguish cDC1s, which were considered CD172a^-/lo^XCR1^+^ and cDC2s were CD172a^hi^XCR1^-/lo^ (64, 65). CD103 was used to further distinguish cDC1 into non-migratory (CD103^lo^) or migratory (CD103^hi^) subsets (39, 47, 64). Plasmacytoid DCs (pDCs) were identified as B220^+^MHC-II^+^Ly6C^+^PDCA-1^+^ (**Supplementary Figures S1A, B**).

**Figure 3:**
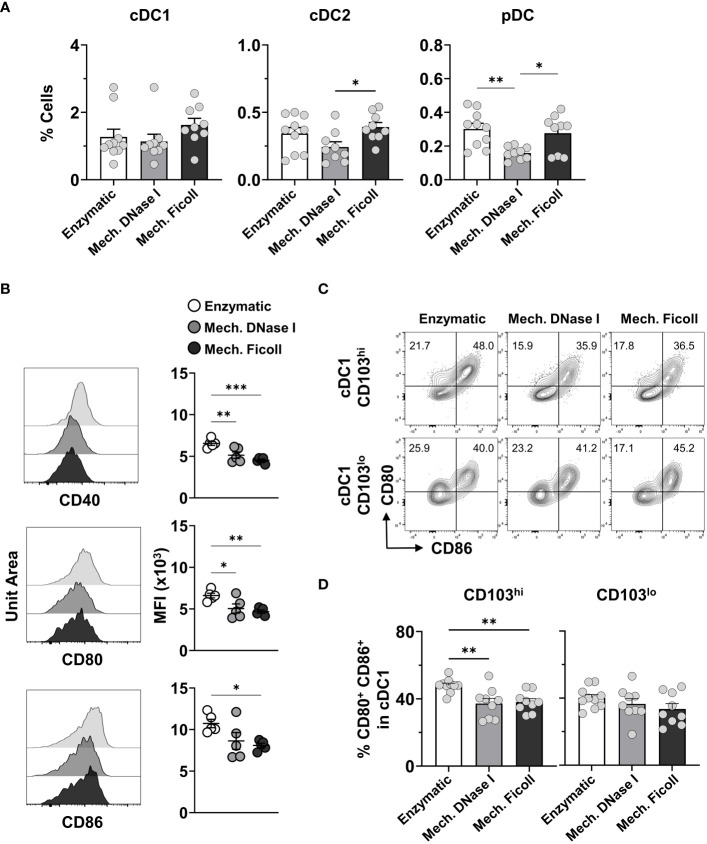
Analysis of distinct dendritic cell populations from neonatal dLNs indicates variations in frequencies and expression of activation markers. **(A)** Frequencies of cDC1, cDC2, and pDCs from neonate dLNs at 2 dpi with RSV processed by indicated protocols. **(B)** Representative stacked histograms and their MFI values for activation markers CD40 (top panel), CD80 (middle panel) and CD86 (lower panel) within the cDC1 populations comparing Enzymatic, Mech. DNase I, and Mech. Ficoll protocols. MFI was representative of two independent experiments. **(C)** Representative contour plots of CD80 and CD86 in the CD103^hi^ (upper panel) and CD103^lo^ (lower panel) cDC1 populations showing the positive frequency of CD80^+^CD86^-^ (top left quadrant) and CD80^+^CD86^+^ (top right quadrant) populations. **(D)** Frequency of CD80^+^CD86^+^ cDC1 by indicated protocols. Data were pooled from two independent experiments with 4-5 mice per experiment. Statistical analysis was performed using a one-way ANOVA Tukey’s multiple comparisons, with *P < 0.05, **P < 0.01, and ***P < 0.005. Error bars represent standard error of the mean.

Lower frequencies of neonatal cDCs and pDCs were measured after processing with the mechanical with DNase I protocol compared to the enzymatic and mechanical with Ficoll protocols (**Figure 3A**). However, the cell counts were similar between the dissociation protocols (**Supplementary Figure S4A**). In the adults, pDCs were statistically higher in frequency after processing the dLN with the enzymatic compared to the mechanical with DNase I (P = 0.001), or Ficoll (P = 0.0139) protocols (**Supplementary Figure S4B**). The cell counts followed the trend in frequencies (**Supplementary Figure S4C**). No difference was observed in frequency or cell count for the adult cDC1 and cDC2 populations between the dissociation protocols.

Costimulatory molecules, such as CD40, CD80 and CD86, are used to measure the activation of APCs. To determine if the dissociation protocols impacted the expression of costimulatory molecules in neonatal mice, we evaluated the MFI of these molecules in the DCs obtained with the three protocols. We analysed heterogeneity of the expression of CD40, CD80 and CD86 between the dissociation protocols in the DC populations. However, the measured MFI for all three costimulatory molecules was significantly higher on the cDC1s isolated with the enzymatic protocol (**Figure 3B**). Furthermore, we assessed the proportion of CD80^+^CD86^+^ cells in the CD103^hi^ migratory and CD103^lo^ non-migratory cDC1 populations. 35-50% of the cDC1s identified in either subset expressed both CD80 and CD86 depending on the protocol (**Figure 3C**), which is indicative of cDC1 activation in the neonatal murine dLN at 2 days post RSV infection (26). The dLN processed with the enzymatic protocol exhibited the highest frequency of CD80^+^CD86^+^ cells in the migratory cDC1 population (**Figure 3D**) when compared to both the mechanical with DNase I (P = 0.0045), or Ficoll protocols (P = 0.0086), consistent with higher MFI measurement for these two costimulatory molecules exhibited in the dLNs processed with the enzymatic protocol, suggesting that the collagenase D containing protocol may result in an over estimation of cDC1 activation.

### 3.4 Monocyte and macrophage subset in the dLN exhibited minimal differences between the dissociation protocols

In response to inflammation both migratory and resident macrophages, as well as circulating and inflammatory monocytes (66–69), can be found in the dLN. These cells have been associated with many functions, such as phagocytosis, antigen presentation, delivery of antigen to resident DCs for cross-presentation, efferocytosis, and cytokine production, which assist in the differentiation of recently activated lymphocytes (68, 70, 71). In the present study, we analyzed a migratory alveolar macrophage (AM)-like Siglec-F^+^ population (Siglec-F^+^CD64^+^Ly6C^+^CD172a^+^), and monocytes (CD11c^-/lo^Ly6C^+^MHC-II^-/lo^) in the dLN. In addition, we measured LN-resident medullary sinus macrophages (MSM, CD169^+^F4/80^+^), subcapsular sinus macrophages (SSM, CD169^+^F4/80^-^), medullary cord macrophages (MCM, CD169^-^F4/80^+^), and T zone macrophages (TZM, CD169^-^F4/80^-^) in the dLN (**Supplementary Figures S1B, C**).

**Figure 4:**
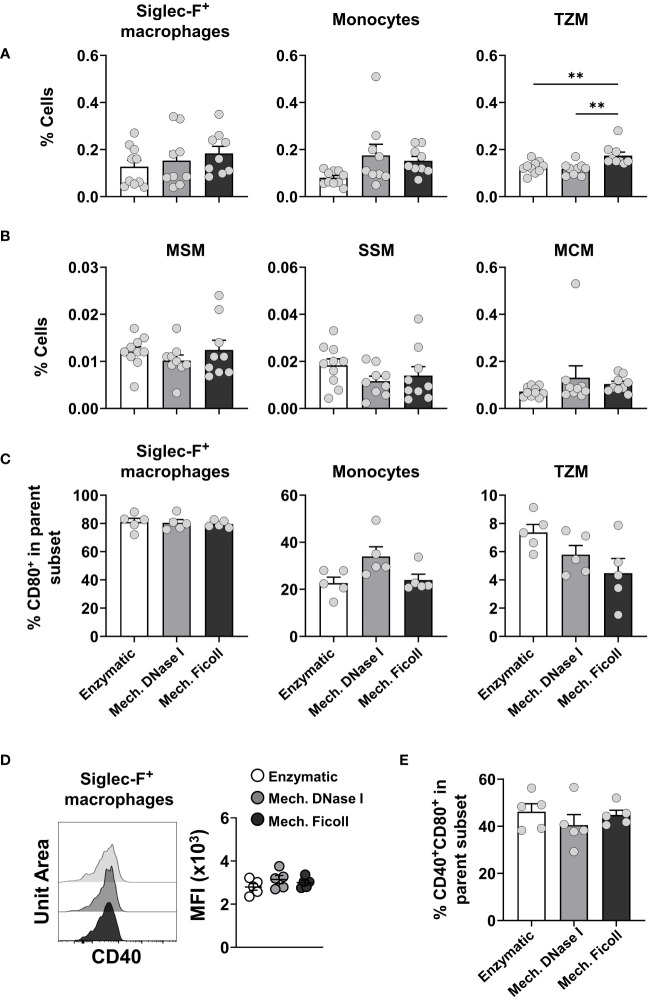
Analysis of monocytes, migratory and LN-resident macrophages from neonatal dLNs. **(A)** Frequency of alveolar macrophage-like Siglec-F^+^ macrophages (Siglec-F^+^CD64^+^Ly6C^+^CD172a^+^) (left panel), monocytes (CD11c^-/lo^Ly6C^+^MHC^-/lo^) (middle panel) and LN-resident T zone macrophages (TZM, CD169^-^F4/80^-^) (right panel). **(B)** Frequency of LN-resident macrophages (from left to right) medullary sinus macrophages (MSM, CD169^+^F4/80^+^), subcapsular sinus macrophages (SSM, CD169^+^F4/80^-^), and medullary cord macrophages (MCM, CD169^-^F4/80^+^). Data were pooled from two independent experiments with 4-5 mice per experiment. **(C)** Frequency of CD80^+^ expressing cells within Siglec-F^+^ macrophages (left panel), monocytes (middle panel) and TZM (right panel). **(D)** Representative stacked histogram of CD40 expression on Siglec-F^+^ macrophages subsets (left panel) and comparison of CD40 MFI on Siglec-F^+^ macrophages between protocols (right panel). **(E)** Frequency of CD40^+^CD80^+^ in Siglec-F^+^ macrophages isolated from neonatal dLNs. Frequency of activated populations were from a representative experiment with 5 individuals per group. Error bars represent standard error of the mean. Statistical analysis was performed using a one-way ANOVA Tukey’s multiple comparisons, with **P < 0.01.

The frequencies of these populations between total live CD45^+^ cells were under 0.5% in all protocols at 2 days post-infection (**Figures 4A, B**). The frequencies of macrophage populations measured in the neonatal dLNs were similar between the dissociation protocols, except for the TZM population which was increased in frequency in the dLN processed with the mechanical Ficoll protocol compared to the mechanical with DNase I (P = 0.0034) and enzymatic (P = 0.0086) protocols (**Figure 4A**). However, TZM cell counts were consistent between the dissociation protocols (**Supplementary Figure S5A**). MSM and SSM were infrequent at 0.04% irrespective of the protocol (**Figure 4B**), and cell counts ranged from 20 to 303 for SSM with the mechanical with DNase I protocol (**Supplementary Figure S5B**). The frequencies of these populations were also low in adult samples, ranging from 0.1% (monocytes, **Supplementary Figure S5C**) to less than 0.005% (MSM, **Supplementary Figure S5D**). The frequencies of the monocyte and macrophage subsets were also very similar in adult dLNs processed with the compared protocols (**Supplementary Figure S5C**), except for the MCM population where the mechanical with DNase I protocol had a greater frequency (**Supplementary Figure S5D**).

To further investigate the activation status of monocyte and macrophage subsets in neonates, we analyzed Siglec-F^+^ macrophages, monocytes, and TZM for costimulatory molecule expression. The frequencies of CD80^+^ cells were similar between the dissociation protocols, with 80% of Siglec-F^+^ macrophages, 20-30% of monocytes, and less than 10% of the TZM expressing CD80 (**Figure 4C**). Siglec-F^+^ macrophages expressed CD40 and the MFI was similar between the dissociation protocols (**Figure 4D**) as was the frequency of CD40^+^CD80^+^ cells (**Figure 4E**). The level of CD86 expression measured on the monocyte and macrophage subsets was very low. Expression levels of the costimulatory molecules were also consistent between these cell populations measured from adult dLNs processed with the compared protocols (data not shown).

Taken together, when comparing processing protocols, our results did not show significant differences in the frequency or cell number of the macrophage and monocyte subsets in the dLN of neonatal and adult mice, except for an increase in the frequency, but not the number of TZM in the neonatal dLN processed with the enzymatic protocol.

### 3.5 Adult, but not neonatal, granulocyte frequencies in the dLN differ after processing with a density gradient separation

**Figure 5:**
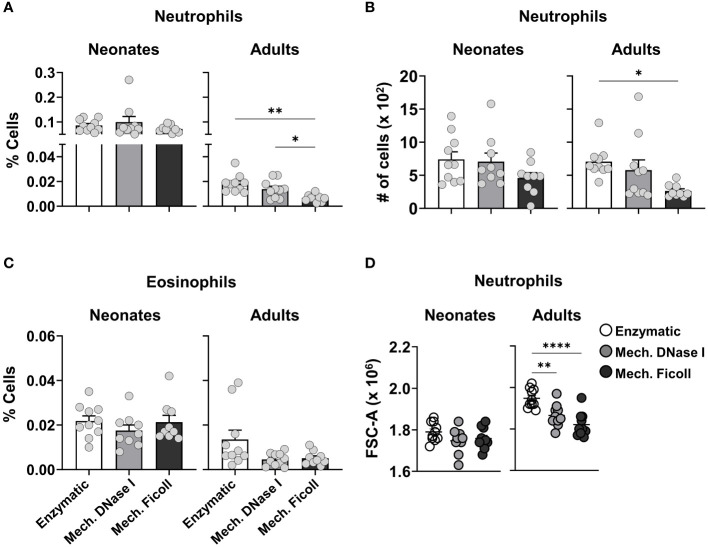
Comparison of granulocytes populations from neonatal and adult dLNs by different dissociation protocols. **(A)** Frequency of neutrophils in neonatal (left) and adult (right) dLNs. **(B)** Cell count of neutrophils in neonatal (left) and adult (right) dLNs. **(C)** Frequency of eosinophils in neonatal (left) and adult (right) samples. **(D)** Forward side scatter values of neutrophils from neonatal (left) and adult (right) dLNs. Data were pooled from two independent experiments with 4-5 mice per experiment. Error bars represent standard error of the mean. Statistical analysis was performed using a one-way ANOVA Tukey’s multiple comparisons, with *P < 0.05, **P < 0.01 and ****P < 0.0001.

Granulocytes, such as neutrophils, basophils, and eosinophils, are characterized by the presence of an abundance of secretory granules in their cytoplasm. These granules increase their cell density. After density gradient separation, frequencies and cell counts of neutrophils were reduced in the adult dLNs (**Figures 5A, B**). Surprisingly, the loss of neutrophils was not observed in the neonatal dLNs processed with a density gradient separation, as the frequencies and cell counts were similar between the dissociation protocols. There was no difference in the frequency of eosinophils across all protocols (**Figure 5C**). To investigate the population of granulocytes remaining after density gradient separation, we further analyzed their forward scatter profile by flow cytometry. We found that the adult neutrophil population exhibited lower forward scatter (FSC) values after density gradient separation (**Figure 5D**). In contrast, the FSC profiles were similar between the neutrophil populations isolated from the neonatal dLNs suggesting that adult neutrophils varied in density and those with greater density were removed.

## 4 Discussion

Technologic advances in multiparameter flow cytometry and single-cell analysis of RNA and proteins enables more complex analysis of immune responses (37, 44, 45, 72). However, the technical approach to processing tissue for single-cell assays can have profound impacts on the data and findings. Dissociation protocols have been designed for different tissues however, age has not previously been considered (45, 46, 51, 56). In this study, we compared three dissociation protocols (24, 26, 45, 61) and their effects on cells isolated from the neonatal murine dLNs 2 days after RSV infection. We found that the three protocols yielded similar numbers and viable cells from the neonatal dLN. We did measure several unexpected differences when comparing cells after processing with three different protocols. A higher yield of CD8^+^ T cells in adult and neonatal dLNs with the enzymatic protocol was observed, suggesting the enzymatic process increases CD8^+^ T cell release from the dLN. However, the MFI of the anti-B220 antibody, a pan-B cell marker, was reduced on B cells, as was CXCR5, a chemokine receptor, after processing dLNs with the enzymatic protocol. This was associated with a lower frequency of B cells compared to the other protocols. The enzymatic protocol was also associated with higher MFI values for the antibodies recognizing CD40, CD80, and CD86 on CD103^hi^ cDC1 compared to the other two protocols suggesting artificially inflated activation of this cell population. Despite, the use of a density gradient in the mechanical protocol, the numbers of neutrophils were similar in the neonatal dLNs between single-cells suspension protocols in contrast to the measured response in adults.

The yield and viability of cells significantly influences the comparison of cell populations measured by flow cytometric analyses. Our results demonstrated similar viability and cell count between the three dissociation protocols for either neonatal or adult dLNs; however, adult dLNs exhibited higher total cell numbers compared to neonatal dLNs. Enzymes including collagenase, elastase, dispase, trypsin, and DNase are considered important for the release of immune cells from solid organs (51, 72, 73) and the skin (74) and have also been applied to LNs (46). The enzymatic protocol used in this study employed collagenase D to release cells from the LN architecture and has previously been published (45). Publications comparing approaches to LN single-cell preparation are limited. While we hypothesized more cells would be obtained with enzymatic processing, our findings did not support this expectation, as cell counts across protocols were very similar. The density gradient separation with Ficoll eliminates non-immune cells and would potentially lead to lower cell counts. Our flow cytometric analysis showed that CD45 negative (non-immune) cells were rare after processing with all three protocols and did not contribute significantly to the total cell count (data not shown). Previous studies have shown that adult LN endothelial cells vary from 1-3 × 10^3^ depending on the inflammatory state (74) and thus do not consistute a large percentage of the LN. Additionally, longer periods of incubation with higher doses of digestion enzymes may also be required to effectively isolate stromal cells (74, 75). As the LN is primarily an immune organ, density gradient separation may remove primarily noncellular material that would not be captured by the FSC and side scatter (SSC) set for flow cytometric analysis of cells. Our data showed that enzymatic digestion with collagenase D did not increase overall cell yields from the neonatal or adult dLN.

Expression of lineage markers and activation markers on cells measured by flow cytometry can be quantified by the MFI. The ability of flow cytometric monoclonal antibodies (mAb) to bind to surface molecules can be impacted by the approach to obtain single-cell suspensions. We observed differences in the frequencies of CD4^+^ and CD8^+^ T cells as well as B cells between the dissociation protocols in both neonatal and adult dLNs. To further understand these differences, we analyzed cell subsets by t-SNE and MFI values. The MFI of B220, employed for B cell identification, exhibited a significant decrease following the enzymatic protocol when compared to the other two processing protocols, for both neonatal and adult murine dLN. B220 (CD45R) is an isoform of CD45 that is expressed on all developmental stages of murine B cells, including activated B cells, making it a frequently used pan-B cell marker in mice (76). We used clone RA3-6B2 from BD Biosciences in our staining panel and we did not test alternative clones to determine if the collagenase disrupts B220 or the binding site of the clone. A published protocol using a longer duration of incubation and a different concentration of collagenase D for digestion of the murine spleen noted reduced expression of CD27 on T cells, but not B220, using the same mAb clone (48). They observed that collagenase-induced loss of CD27 expression on CD3^+^ and CD20^+^ cells was recovered after 8 hrs of resting incubation. We also measured a reduction in CXCR5 MFI, which is a chemokine receptor that binds to CXCL13 expressed by follicular DCs and helper T cells directing B cells into the follicles of secondary lymphoid organs (77). CXCR5 reduction after enzymatic digestion has not previously been noted, though chemokine receptors are not included in all flow panels. Together our findings and those of others suggest that collagenase, as well as dispase, may reduce the ability to measure some surface molecules with flow cytometric mAb.

Despite changes in the frequencies of the larger lymphocyte populations, we observed minimal variations in the frequencies or cell counts of neonatal dendritic cell subsets and macrophage populations between the protocols. The only exception was that the frequency and cell number of plasmacytoid dendritic cells (pDCs) was higher after enzymatic digestion of adult dLNs compared to the other two protocols. This trend was similar in neonatal dLNs. Our data suggest that mechanical dissociation using frosted slides is adequate for releasing conventional dendritic cells (cDCs) and macrophages from the adult and neonatal murine lymph nodes, but less so for pDCs.

Costimulatory molecule expression of CD40, CD80 and CD86 were evaluated on neonatal DCs and macrophages, as they can be used as phenotypic and activation markers in these cell populations. The MFI associated with CD40, CD80 and CD86 was highest on the cDC1 populations in the neonatal dLN and was 2-fold higher after enzymatic digestion compared to the other protocols. We further divided the populations into migratory (CD103^hi^) and non-migratory (CD103^lo^) subsets to characterize the increased MFI expression of CD80 and CD86. Furthermore, the frequency of the CD80 and CD86 double positive population was higher in the migratory cDC1 population after enzymatic digestion. The increase of CD80 or CD40 was not observed in Siglec-F^+^ macrophages, indicating that this increase was not due to direct interactions of collagenase D with the markers, but rather sensitivity of neonatal migratory cDC1s. In addition, adult cDC1s exhibited a trend toward increased CD86 expression after enzymatic digestion (data not shown). Although not statistically significant, this trend suggests that collagenase D digestion may have a greater impact on neonatal cDC1, making data between adults and neonates less comparable after collagenase digestion. Our data suggest that the enzymatic protocol used here may alter the expression of costimulatory molecules on neonatal cDC1s in the LN. A recent human study found that warm enzymatic digestion activated placental macrophages. While there were no significant differences in gene expression between biopsies stored on ice and biopsies stored at 37°C, warm enzymatic digestion increased the expression of proinflammatory cytokines in placental macrophages compared to cold mechanical dissociation (78).

Density gradients can be used to selectively isolate cells based on granularity (57–60). Due to the higher granularity and density of neutrophils and eosinophils, we expected lower recovery of these cells after dLNs were processed with the mechanical protocol with density gradient separation compared to the other protocols. Our protocol uses a Ficoll-based cell separation media with at density of 1.086 g/ml. Leukocytes have different densities and have been separated based on these qualities for many decades (79, 80). Neutrophils and other granulocytes have a greater density than lymphocytes and are considered to be best isolated at a density of 1.10-1.119 g/ml (81). Interestingly, single-cell suspensions from the adult dLN showed reduced numbers of granulocyte populations after processing with a density gradient, whereas the number of neutrophils isolated from the neonatal dLN showed very similar numbers and frequencies between protocols. Low density neutrophils (LDN) have been identified in human neonates at higher proportions than in healthy adults (82–84), but inflammation can increase LDN in adults as well (85). Due to their lower density, they are not removed by density gradients standardly used to isolate lymphocytes (84). Further surface molecule evaluation would be required to characterize the neutrophils measured in our study (84, 86). The antibody panel used in this study focused on the phenotype and activation of lymphocytes and myeloid APCs, thus contained few markers to characterize granulocytes. We were however able to show that neutrophils isolated from the adult dLN with the enzymatic protocol had higher FSC compared with the mechanical protocols, but the FSC was lower for the neonatal neutrophils processed with all three protocols, suggesting that neonatal neutrophils in general had a lower density.

In summary, our results demonstrate that the dissociation methods evaluated in this study influenced the frequency and phenotype of the lymphocyte populations as well as expression of activation markers of DC populations. However, in this study the enzymatic treatment did not increase the yield of most of the myeloid populations analyzed in the dLN from neonatal or adult mice.

## Data availability statement

The raw data supporting the conclusions of this article will be made available by the authors, without undue reservation.

## Ethics statement

The animal study was approved by the USU Institutional Animal Care and Use Committee. The study was conducted in accordance with the local legislation and institutional requirements.

## Author contributions

JD: Formal analysis, Investigation, Methodology, Resources, Visualization, Writing – original draft, Writing – review & editing. AZ: Investigation, Writing – original draft, Writing – review & editing. PN: Investigation, Writing – review & editing. KE: Investigation, Writing – review & editing. JK: Formal analysis, Visualization, Writing – review & editing. ZL: Formal Analysis, Investigation, Writing – original draft, Writing – review & editing. AM: Conceptualization, Funding acquisition, Project administration, Resources, Supervision, Writing – original draft, Writing – review & editing

## Author disclaimer

The contents of this publication are the sole responsibility of the author(s) and do not necessarily reflect the views, opinions, or policies of Uniformed Services University of the Health Sciences (USU); National Institutes of Health or the Department of Health and Human Services; the Henry M. Jackson Foundation for the Advancement of Military Medicine, Inc.; the Department of Defense (DoD); the U.S. Air Force, the U.S. Army, U.S. Navy, the U.S. military at large, or the U.S. Department of Defense. Title 17 U.S.C. 105 provides that ‘Copyright protection under this title is not available for any work of the United States Government.’ Title 17 U.S.C. 101 defines a “United States Government work” as a work prepared by a military service member or employee of the United States Government as part of that person’s official duties. Mention of trade names, commercial products, or organizations does not imply endorsement by the U.S. Government.
