# Supplementary material for: Dissociation protocols influence the phenotypes of lymphocyte and myeloid cell populations isolated from the neonatal lymph node
Source: Front Immunol. 2024 May 1;15:1368118. doi: 10.3389/fimmu.2024.1368118 (PMC11097666; doi:10.3389/fimmu.2024.1368118)
Supplement: Supplementary file 1 [file DataSheet_1.pdf]

Supplementary Table 1. Antibody Panel for Spectral Flow Cytometry. Antibodies were titrated by lot number before use in experiments.

Marker	Fluorochrome	Clone	Manufacturer	Cat. No.
B220 (CD45R)	BUV395	RA3-6B2	BD	563793
Live/Dead	Blue	-	Thermo Fisher	L34962
CD172 (SIRPα)	BUV615	P84	BD	751214
CD80	BUV661	16-10A1	BD	741515
CD3	BUV737	145-2C11	BD	612771
CD86	BUV805	GL1	BD	741946
CCR7	BV421	4B12	BioLegend	120120
CD40	BV480	44643	BD	746351
XCR1	BV510	ZET	BioLegend	148218
CD11c	BV570	N418	BioLegend	117331
CD169 (Siglec-1)	BV605	3D6.112	BioLegend	142413
F4/80 (Ly71)	BV650	BM8	BioLegend	123149
I-A/I-E	BV711	M5/114.15.2	BioLegend	107643
CD4	BV750	GK1.5	BioLegend	100467
Ly6C	BV785	HK1.4	BioLegend	103059
CXCR5	FITC	L138D7	BioLegend	145520
CD8a	PerCP	53-6.7	BioLegend	100732
CD24	PerCP Cy5.5	M1/69	BioLegend	101824
PDCA-1	PE	129c1	BioLegend	127103
CD103	PE Dazzle 594	2E7	BioLegend	121430
CD11b	PE Cy5	M1/70	BioLegend	101210
Siglec-F	PE-Vio770	REA789	Miltenyi	130-112-176
CD64	AF647	X54-5/7.1	BioLegend	139322
Ly6G	AF700	1A8	BioLegend	127622
NK1.1	APC Cy7	PK136	BioLegend	108724
CD45	APC Fire810	30F11	BioLegend	103174

Figure S1

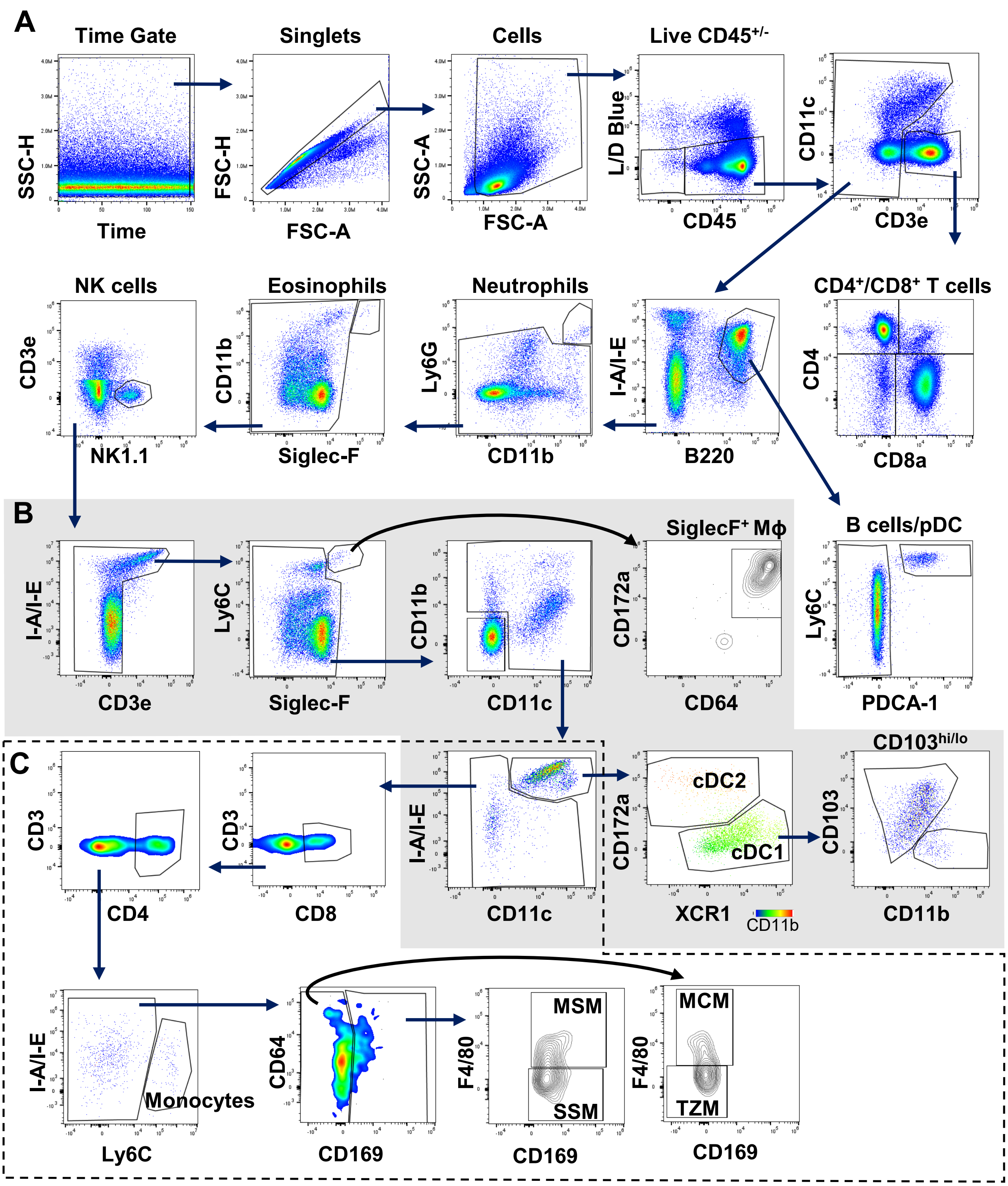
A
Time Gate
Singlets
Cells
Live CD45+/-
SSC-H
Time
FSC-H
FSC-A
SSC-A
L/D Blue
CD45
CD11c
CD3e
NK cells
Eosinophils
Neutrophils
CD4+/CD8+ T cells
NK1.1
Siglec-F
CD11b
Ly6G
I-A/I-E
B220
CD4
CD8a
B
SiglecF+ Mϕ
B cells/pDC
Ly6C
CD11c
CD172a
CD64
PDCA-1
C
CD103hi/lo
CD3
CD8
cDC2
cDC1
XCR1
CD103
Monocytes
CD169
F4/80
MSM
SSM
MCM
TZM

Supplemental Figure S1. Gating strategy for identification and analysis of immune cells from the mLNs of neonates and adults at 2dpi. Representative gating on a neonatal sample processed with the enzymatic protocol. Single-cell suspensions from each protocol were stained with fluorescently labeled antibodies and acquired on a flow cytometer. **(A)** Live $CD45^{+}$ and $CD45^{-}$ cells were identified after gating out flow stream irregularities, doublets and cellular debris. CD3 positive and negative cells were identified. $CD3^{+}$ cells were further gated in $CD4^{+}$ or $CD8^{+}$. $CD3^{-}$ cells were gated B220 vs I-A/I-E to identify B cells (PDCA-1^{-}) and pDCs (PDCA-1^{+}), whereas non-$B220^{+}$I-A/I-E^{+} events were gated for neutrophils ($CD11b^{+}Ly6G^{hi}$), and eosinophils ($CD11b^{+}$Siglec-F^{hi}). NK cells were gated from $CD3^{-}NK1.1^{+}$. **(B)** Gating strategy for Siglec-F^{+} macrophages and cDCs. Events from NK1.1 negative gate as shown in (A) were further gated to remove residual $B220^{+}$ and $CD3^{+}$ cells, and gated Ly6C vs. Siglec-F. $Ly6C^{hi}$, Siglec-F^{+} events were further gated as $CD172a^{+}CD64^{+}$ alveolar macrophage-like Siglec-F^{+} macrophages. $CD11c^{-}CD11b^{-}$ cells were removed from the remaining cells in the Ly6C vs. Siglec-F. $CD11c^{+}CD11b^{+}$ cells were gated to distinguish $CD11c^{hi}$I-A/I-E^{hi} cDCs. cDCs were gated into $CD172a^{+}XCR1^{-/lo}$ cDC2 and $CD172a^{-/lo}XCR1^{+}$ cDC1. The latter population was further gated to define $CD103^{hi}$ migratory cDC1 and $CD103^{lo}$ non-migratory cDC1 populations. **(C)** Gating strategy for monocytes and LN-resident macrophages. Cells previously gated in (B) as non-$CD11b^{hi}CD11c^{hi}$ were selected and remaining $CD3^{+}CD8^{+}$ and $CD3^{+}CD4^{+}$ events were removed. Remaining cells were gated in I-A/I-E vs. Ly6C as $Ly6C^{+}$ monocytes and $Ly6C^{-}$ population. Ly6C negative cells were plotted in CD64 vs CD169 to gate cells $CD169^{-}$ and $CD169^{+}$ cells. $CD169^{+}$ cells were further gated to separate medullary sinus macrophages (MSM, $CD169^{+}F4/80^{+}$) and subcapsular sinus macrophages (SSM, $CD169^{+}F4/80^{-}$) $CD169^{-}$ cells were further gated to distinguish medullary cord macrophages (MCM, $CD169^{-}F4/80^{+}$) and T zone macrophages (TZM, $CD169^{-}F4/80^{-}$).

Figure S2

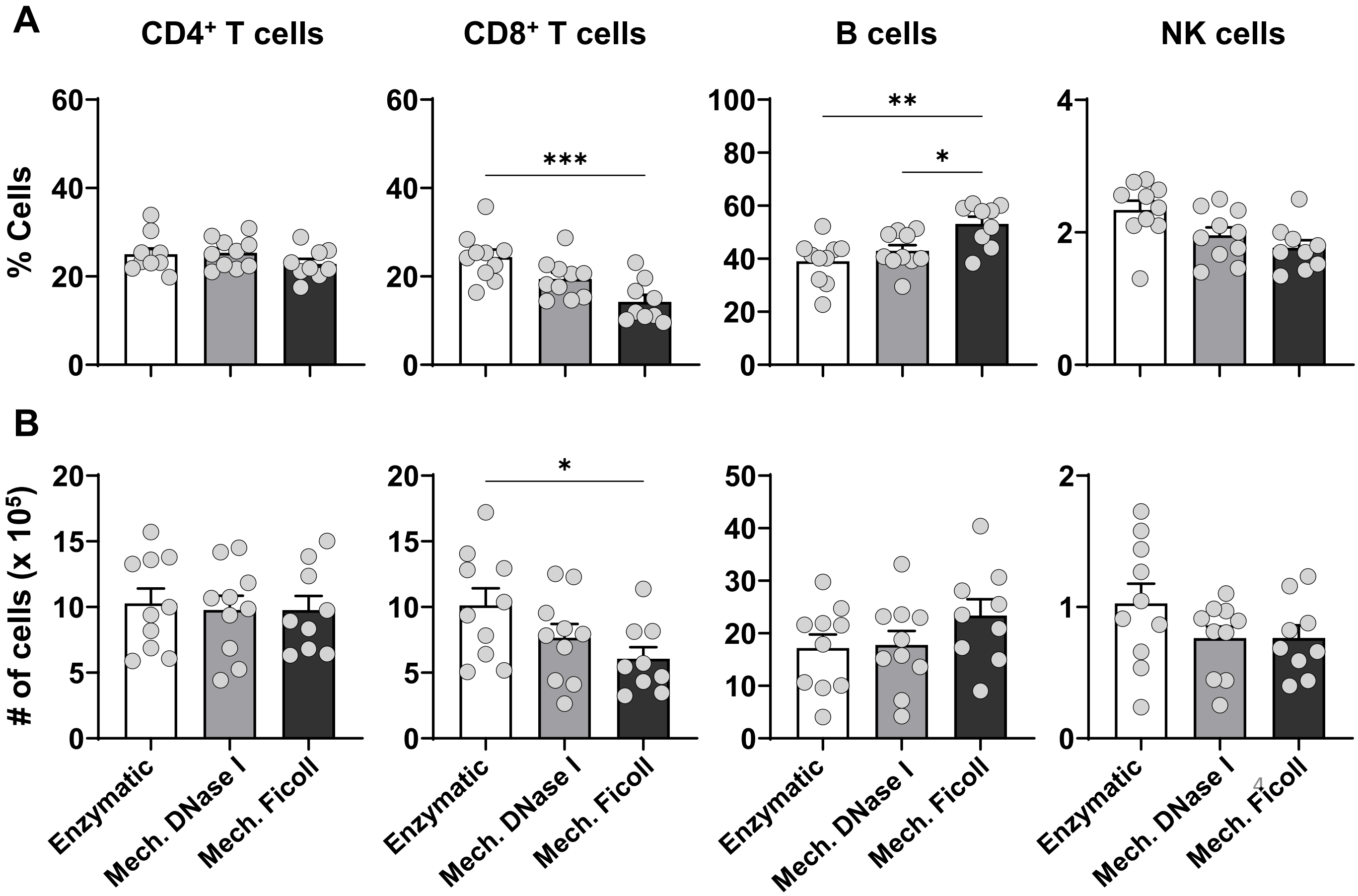

Supplemental Figure S2. The frequencies and cell number of lymphocyte populations in adult mLNs at 2dpi. Frequencies **(A)** and cell number **(B)** of $CD4^+$ and $CD8^+$ T cells, B cells and NK cells from adult mLNs at 2 dpi. Data was pooled from two independent experiments with 9-10 animals per group. Error bars represent standard error of the mean. Statistical analysis was performed using a one-way ANOVA Tukey's multiple comparisons, with $^*P < 0.05$, $^{**}P < 0.01$, and $^{***}P < 0.005$.

Figure S3

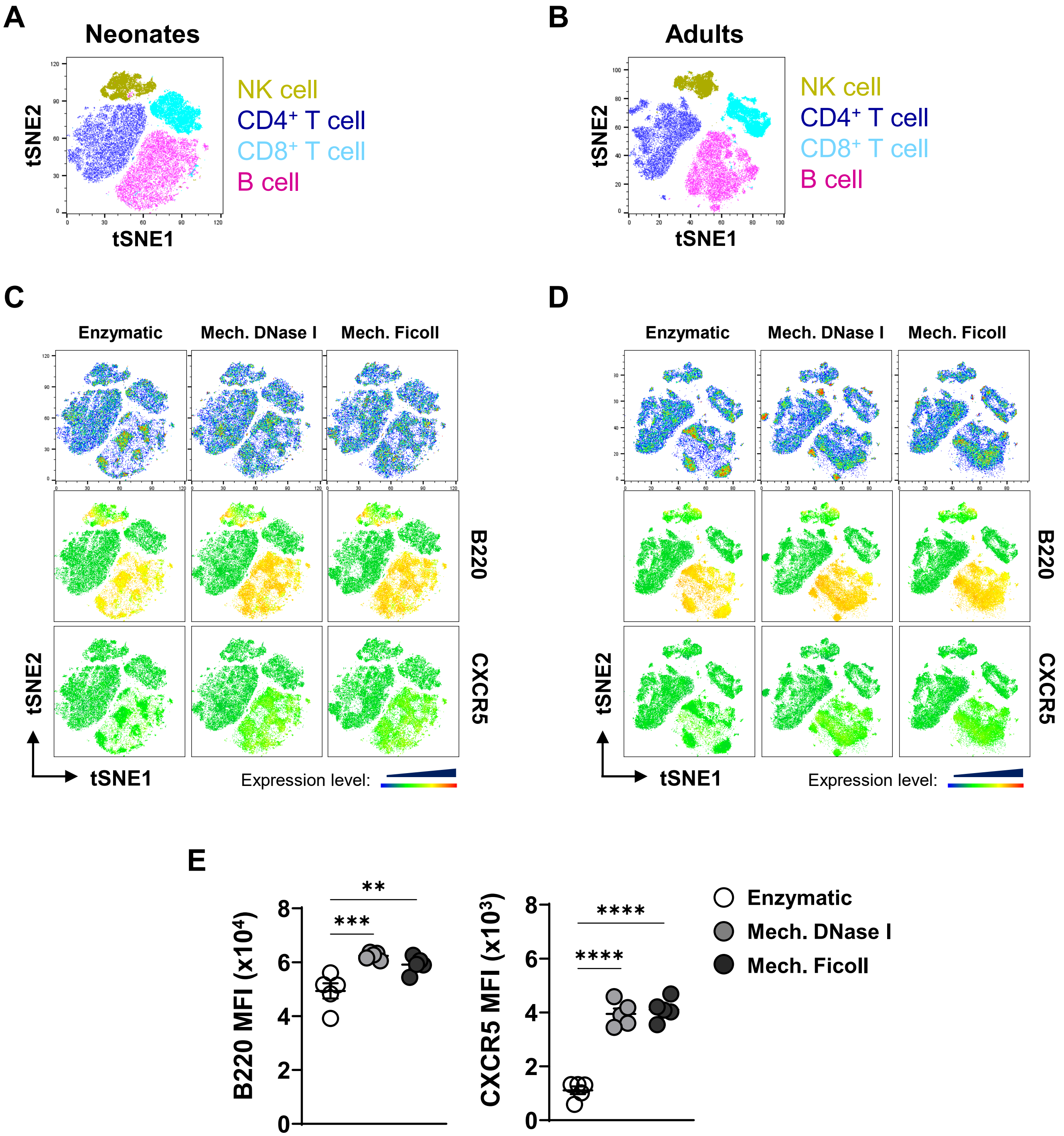

Supplemental Figure S3. Cell surface molecules of lymphocyte populations in mLNs at 2dpi. **(A and B)** tSNE analysis of lymphocyte populations of concatenated samples from neonates (A) or adults (B) with the three protocols showing NK cells, $CD4^+$ T cells, $CD8^+$ T cells and B cells, which were gated in supplemental figure S1A. **(C and D)** Comparison by tSNE analysis of lymphocyte subsets from the Enzymatic (left), Mech. DNase I (central) or Mech. Ficoll (right) protocols from concatenated neonatal (C) or adult (D) mLN samples. Top panels represent distribution of populations, middle and lower panels represent expression of B220 and CXCR5 as heat maps, respectively. **(E)** Expression of B220 and CXCR5 on B cell subsets in adult mice shown as MFI values comparing protocols from Enzymatic (open), Mech. DNase I (light gray) and Mech. Ficoll (dark gray) groups. Gating and analysis were performed with FlowJo v10.8.1. Error bars represent standard error of the mean. Statistical analysis was performed using a one-way ANOVA Tukey's multiple comparisons, with $*P < 0.05$, $**P < 0.01$, $***P < 0.005$, and $****P < 0.0001$.

Figure S4

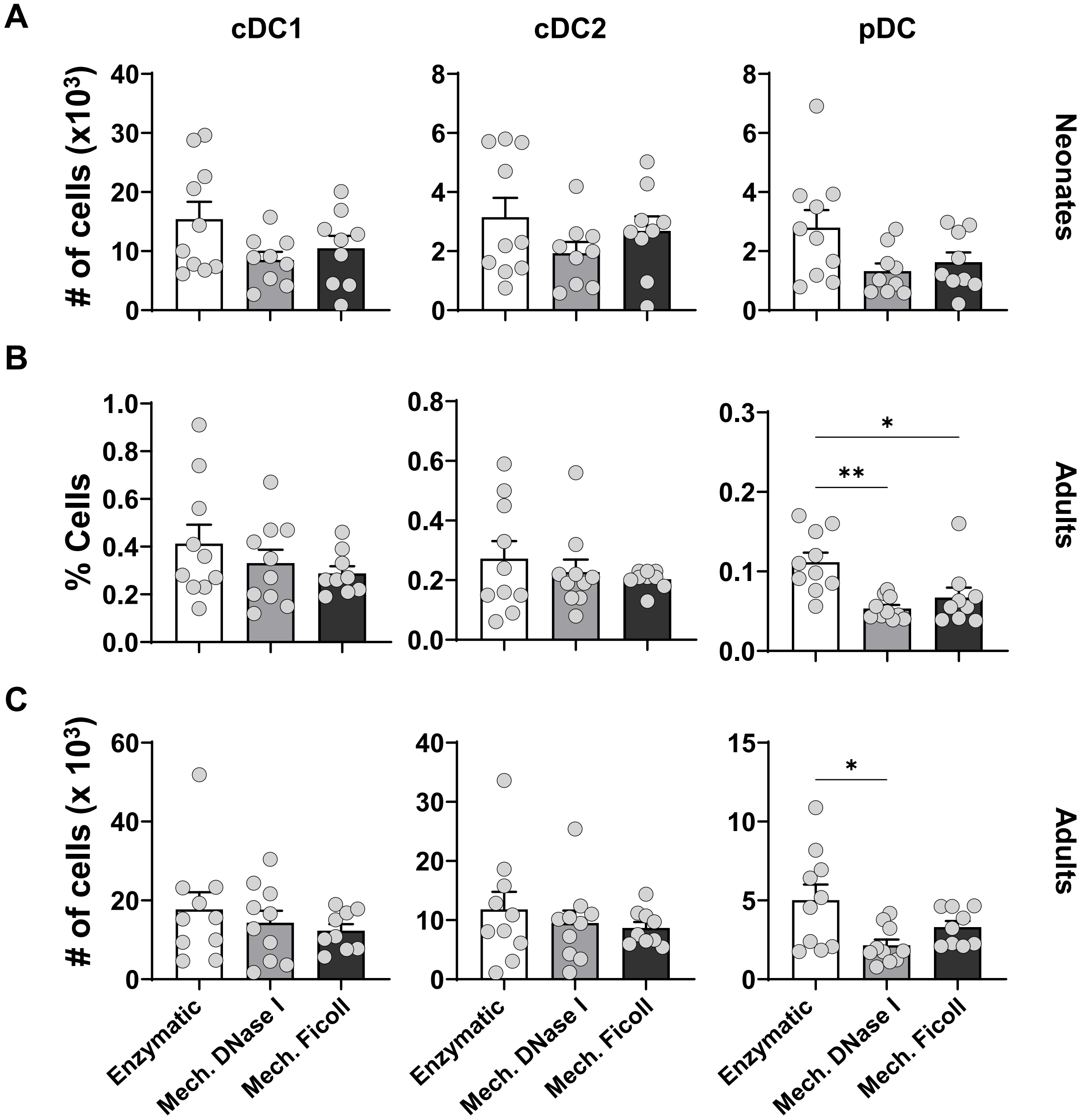

Supplemental Figure S4. Frequencies and cell number of dendritic cell populations in mLNs at 2 dpi. **(A)** Cell number of cDC1s, cDC2s, and pDCs in neonate mLN samples. **(B)** Frequencies of cDC1s, cDC2s, and pDCs in adult mLN samples. **(C)** Cell number of cDC1s, cDC2s, and pDCs in adult mLN samples Data represents two independent experiments with 4-5 mice per group per experiment. Gating and analysis were performed with FlowJo v10.8.1. Error bars represent standard error of the mean. Statistical analysis was performed using a one-way ANOVA Tukey's multiple comparisons, with $^{*}P < 0.05$, and $^{**}P < 0.01$.

Figure S5

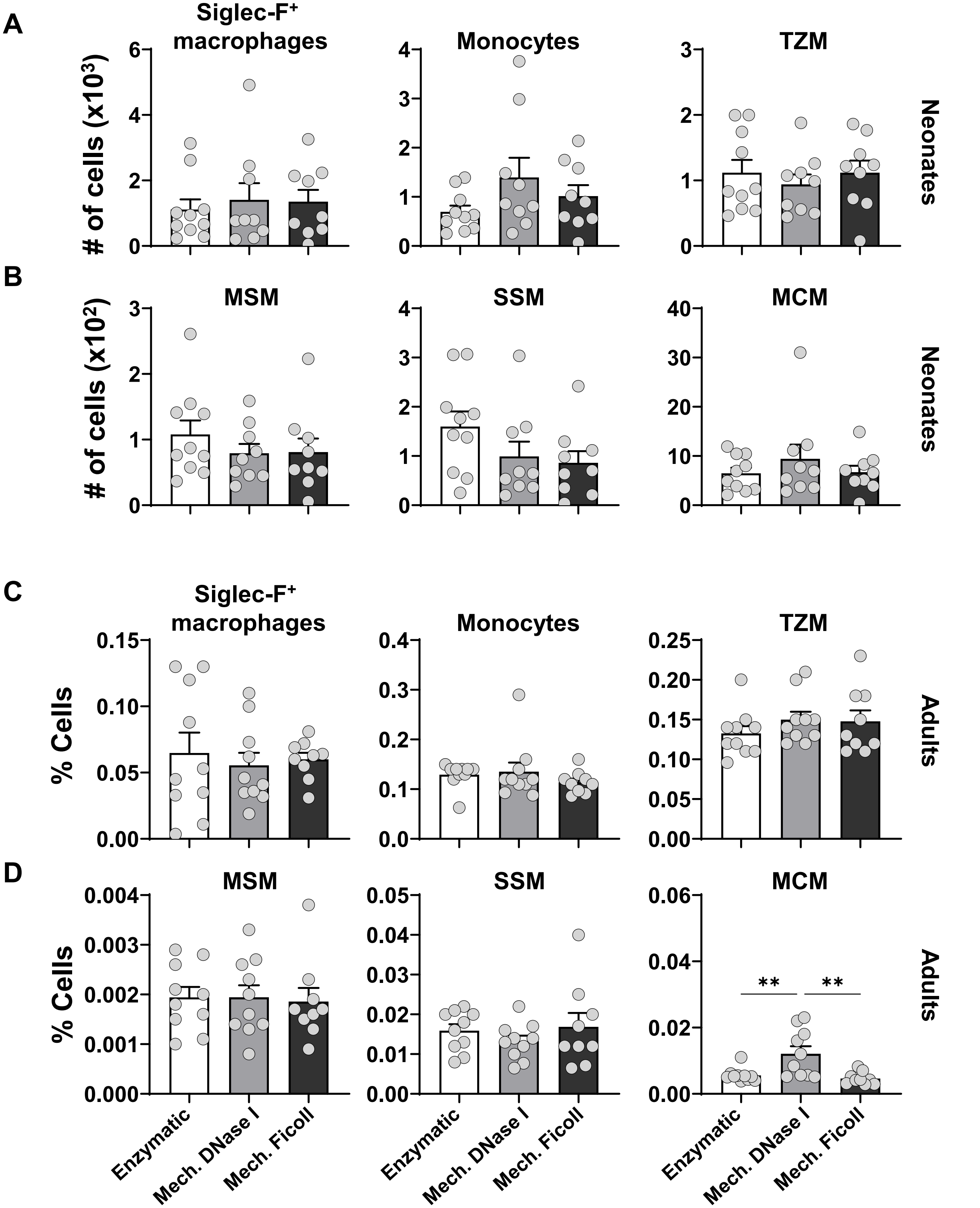

Supplemental Figure S5. Frequencies and cell number of monocytes and LN-resident macrophages. Cell number of neonatal **(A)** Migratory Siglec-F^{+} macrophages, monocytes, and LN-resident TZM, as well as **(B)** LN-resident MSM, SSM and MCM (from left to right). Frequency of adult **(C)** Migratory Siglec-F^{+} macrophages (left), monocytes (center), and TZM (right panel), and **(D)** LN-resident MSM, SSM and MCM (left, middle and right panels, respectively). Data is shown as a compilation of two independent experiments, with 9-10 mice total per group. Error bars represent standard error of the mean. Statistical analysis was performed using a one-way ANOVA Tukey's multiple comparisons, with $*P < 0.05$, and $**P < 0.01$.
